# Comparative secretome analysis of *Rhizoctonia solani* isolates with different host ranges reveals unique secretomes and cell death inducing effectors

**DOI:** 10.1038/s41598-017-10405-y

**Published:** 2017-09-05

**Authors:** Jonathan P. Anderson, Jana Sperschneider, Joe Win, Brendan Kidd, Kentaro Yoshida, James Hane, Diane G. O. Saunders, Karam B. Singh

**Affiliations:** 1 CSIRO Agriculture and Food, Floreat, Western Australia Australia; 2 The UWA Institute of Agriculture, University of Western Australia, Crawley, Western Australia Australia; 3 The Sainsbury Laboratory, Norwich, UK; 4 The John Innes Centre, Norwich, UK; 5 Present Address: Curtin University, Bentley, Western Australia Australia

## Abstract

*Rhizoctonia solani* is a fungal pathogen causing substantial damage to many of the worlds’ largest food crops including wheat, rice, maize and soybean. Despite impacting global food security, little is known about the pathogenicity mechanisms employed by *R. solani*. To enable prediction of effectors possessing either broad efficacy or host specificity, a combined secretome was constructed from a monocot specific isolate, a dicot specific isolate and broad host range isolate infecting both monocot and dicot hosts. Secretome analysis suggested *R. solani* employs largely different virulence mechanisms to well-studied pathogens, despite in many instances infecting the same host plants. Furthermore, the secretome of the broad host range AG8 isolate may be shaped by maintaining functions for saprophytic life stages while minimising opportunities for host plant recognition. Analysis of possible co-evolution with host plants and *in-planta* up-regulation in particular, aided identification of effectors including xylanase and inhibitor I9 domain containing proteins able to induce cell death *in-planta*. The inhibitor I9 domain was more abundant in the secretomes of a wide range of necrotising fungi relative to biotrophs. These findings provide novel targets for further dissection of the virulence mechanisms and potential avenues to control this under-characterised but important pathogen.

## Introduction

*Rhizoctonia solani* Kühn is a species complex of fungal pathogens causing substantial impact on the production of a wide range of plants important to humanity. *R. solani* causes agriculturally significant disease on all of the 15 largest food crops worldwide (Supplementary Table S1) as well as many other important food, feed and fibre crops^1^. The vast agronomic impact of *Rhizoctonia* is largely due to the lack of effective genetic resistance in most of the crops and limitations in chemical and cultural controls, particularly in developing countries^2^. Despite the economic and social impact of the pathogen, relatively little is known about how it causes disease. The large genetic distance between *R. solani*, in the Agaricomycetes class of Basidiomycota, and other well-characterised necrotrophic fungal pathogens of plants, which are mostly in the Dothideomycetes class within Ascomycota, further obscures efforts to understand the molecular mechanisms that underpin its pathogenicity.

Recent efforts to sequence and analyse the genomes of several *R. solani* isolates^3–9^ have presented opportunities to begin to tease apart the genetic determinants of pathogenicity. Genomic analysis of a rice infecting AG1-IA isolate identified three proteins eliciting a cell death response when infiltrated into rice plants^9^. One of these, a protein containing a protease inhibitor I9 domain, was shown to elicit differential responses when infiltrated into different rice cultivars suggesting it may contribute to differential pathogenicity of the isolate on these hosts^9^.

To establish infection, fungi re-program plant cells and disrupt the plant immune system by secreting proteins known as effectors^10^. Fungal effectors function either in the plant apoplast or are translocated into the plant cytoplasm^11^. The mechanisms by which fungal effectors are delivered into the host cell are poorly understood and fungal effector prediction is less developed than bacterial and oomycete pathogens where characterised motifs are often used to identify candidate effectors^12^. Biotrophic and hemibiotrophic fungal pathogens employ specialized structures such as haustoria to colonise the plant. Purification of these tissues and analysis of gene expression and proteins can provide an avenue for the identification of higher confidence candidate effectors^13^. Necrotrophic pathogens, such as *R. solani*, often lack these specialised feeding structures, making physical isolation of high confidence effectors a challenge.

Despite the lack of consensus sequence motifs or physical purification methods, unifying features of fungal effector proteins can be exploited to predict likely candidate effectors in newly sequenced pathogen genomes. Furthermore, subclasses of effectors can be predicted based on more specific characteristics. For example, effectors functioning in the plant apoplast have been linked to a high cysteine content and/or small size^14^ while in certain fungal pathogens, effectors are linked to location in gene clusters, gene-sparse regions, areas rich in repetitive DNA and/or dispensable chromosomes^12^. Moreover, the presence of diversifying selection in a secreted fungal protein can indicate a likely interaction with, or detection by, plant protein(s) and computational methods have been employed to identify candidate effectors using these criteria^15^.

In this study, we compare the predicted secretome of three important *R. solani* isolates. The AG8 WAC10335 isolate has a broad host range including both monocots and dicots. Hosts of particular relevance to agriculture are wheat and barley, canola and various legume species^16^. To identify secreted proteins that may relate to the ability to cause disease on monocots or dicots, we compared the AG8 secretome to isolates restricted to either dicots or monocots. The AG3 Rhs1AP isolate has a narrow host range restricted to solanaceous plants including potato, eggplant, pepper, and tomato^3^. The AG1-IA isolate^17^ was isolated from rice in southern China. Although other members of this anastomosis group can also cause disease on soybean or maize, it is thought that rice- and soybean-infecting populations of AG1-IA represent subdivided populations with low genetic exchange that are becoming increasingly divergent due to host specialisation^18^. We predicted and analysed a combined AG8, AG1-IA and AG3 secretome for effector-like characteristics and discovered that it does not resemble that of other well-characterised necrotising plant pathogens. Through our in-depth analysis of the characteristics of the secreted proteins, including many characteristics not previously explored in *R. solani*, we found that the presence of diversifying selection and *in-planta* up-regulation in particular, were useful characteristics for identification of potential effectors. Two such proteins, a xylanase and an inhibitor I9 domain containing protease, were sufficient to induce plant cell death when expressed in *N. benthamiana* suggesting a potential role in the pathogenesis of this necrotrophic pathogen. Inhibitor I9 domain containing proteins were observed to be more prevalent in the secretomes of a range of necrotising fungi compared to biotophic fungi. Together, these findings should help the characterisation of effectors and advance our understanding of the mechanisms of pathogenesis in necrotrophic fungi.

## Results

### Broad host range *R. solani* secretes fewer proteins than other filamentous pathogens

To understand the functions contained within the secreted proteins of *R. solani* and how they may relate to host range, the secretomes of five available *R. solani* isolates were predicted. The AG8, AG1-IA and AG3 isolates all possessed a similar small proportion of secreted proteins (Table 1, Supplementary Data S1) whereas the isolates AG2-2IIIB (a pathogen of sugarbeet) and AG1-IB (a pathogen of lettuce and vegetables) possessed higher proportions of predicted secreted proteins (Table 1, Supplementary Data S2), a finding consistent with Wibberg *et al*.^8^. The secretome prediction methods used herein allowed direct comparison with secretome predictions previously carried out for 44 other fungal species^19^. The size of the AG8, AG1-IA, AG3 predicted secretomes were substantially lower than the average calculated from 17 other fungal plant pathogens and more in line with the average from 17 non-pathogenic species (Table 1, the individual species used for calculation of averages are detailed in Lin *et al*.^19^). To investigate the conservation of secreted proteins between a broad host range isolate and isolates restricted to monocots or dicots this study focused on the AG8 (broad host range including dicots and monocots), AG3 (dicot specific) and AG1-IA (monocot specific) isolates. Redundancy within the secretome was reduced by grouping proteins containing high sequence similarity using the CD-hit algorithm (90% identity) to produce a final list of 2,209 unique proteins that represent the combined secretome of the three isolates (Supplementary Data S3). To further structure the combined secretome, TribeMCL was used to create groups of proteins sharing sequence similarity termed “tribes”. In total 774 tribes were identified, of which 179 tribes had three or more members, with the largest tribe, Tribe 1, containing 62 proteins. The allocation of secretome proteins to tribes is detailed in Supplementary Data S3.

**Table 1:** The proportion of genes predicted to be secreted from fungal isolates.

Isolate	Number of gene models predicted to be secreted	Percent of gene models predicted to be secreted
*R. solani* AG8^4^	762	5.4%
*R. solani* AG1-IA^9^	430	4.0%
*R. solani* AG3^3^	1078	5.0%
*R. solani* AG2-2IIIB^8^	1066	9.0%
*R. solani* AG1-IB^5^	824	6.7%
Plant pathogens average^#^		7.4%^1^
Non-pathogens average^#^		5.3%^2^
*Rhizophagus irregularis* (mycorrhiza)^#^		1%
Animal pathogens average^#^		4.7%^3^

^#^Secretome predictions from^19^ using the same prediction method. ^1^Derived from analysis of 17 fungal pathogens of plants. ^2^Derived from analysis of 17 fungal non-pathogens. ^3^Derived from analysis of 10 fungal pathogens of animals.

### Dicot infecting isolates encode similar secretomes while monocot infecting isolates are more diverse

**Figure 1:**
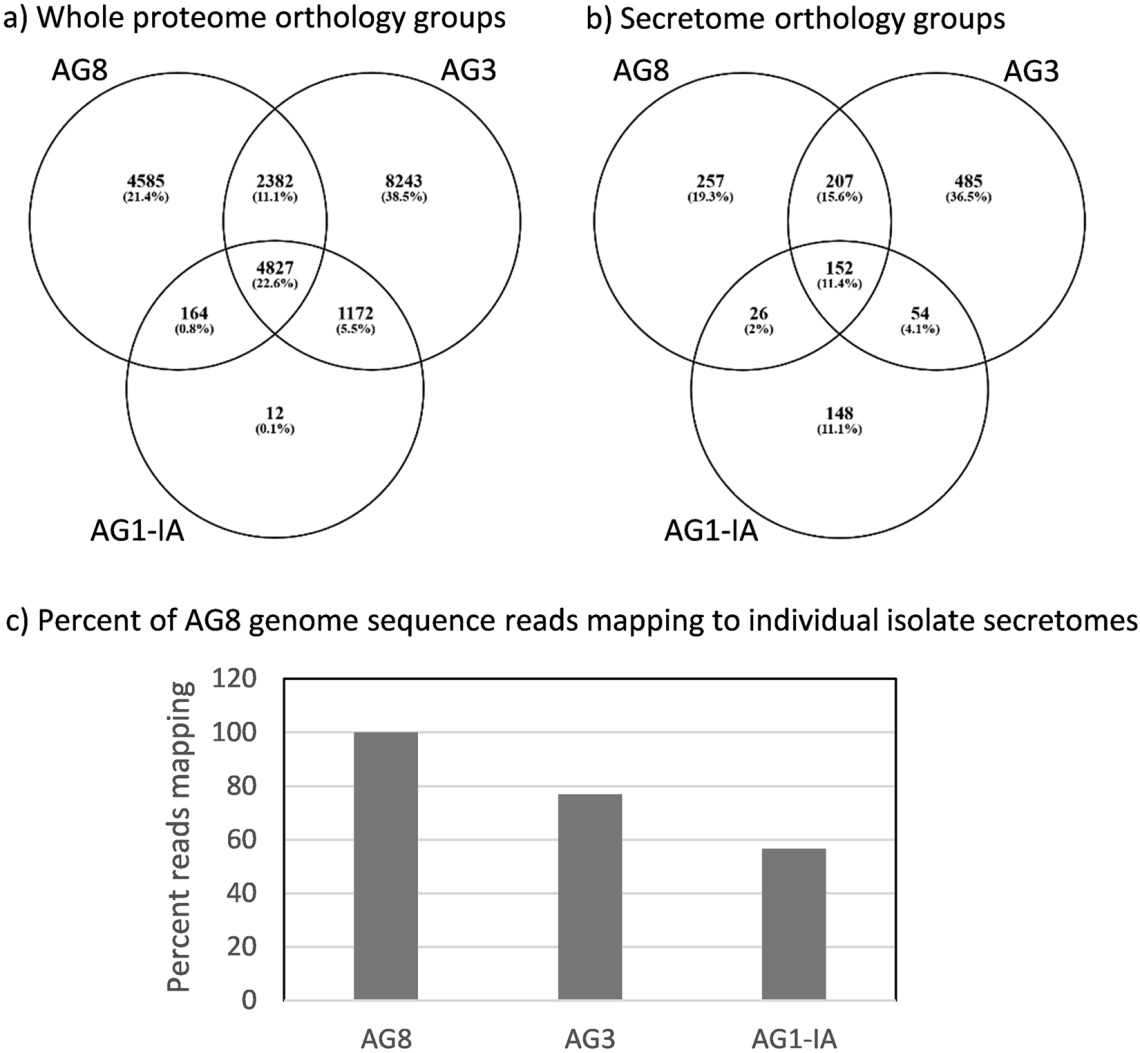
Similarity between AG8, AG1-IA and AG3. Proteins from the entire proteomes (**a**) or secretomes (**b**) from AG8, AG1-IA and AG3 were grouped into orthology groups (OGs) using Orthofinder and shared and isolate-specific OGs plotted in Venn diagrams. The number in each sector represents the number of OGs with members from those isolates and the percentage is the percent of all OGs. (**c**) The percent of genes to which at least one AG8 genomic DNA Illumina read mapped, indicating the potential conservation of the gene even though the complete gene may not have been assembled in the draft genome.

To explore the degree of similarity between the secretomes, two approaches were taken. Firstly, sequence based orthology grouping was performed using Orthofinder^20^ for both the whole proteome and secretome of AG8, AG1-IA and AG3 (Fig. 1). The genome assemblies for the three isolates employed various techniques to account for ploidy/heterozygocity^3, 4, 9^, and thus direct comparisons of protein family size should be interpreted with caution and were not pursued in this study. Instead we focussed on the grouping of proteins into orthology groups, which was not influenced by the number of representative proteins from a particular isolate. Secondly, to determine the number of AG8 genes present or absent between the isolates we also mapped the AG8 genomic reads to the genes constituting the combined secretome. Both approaches found the highest overall similarity was observed between the dicot infecting isolates AG8 and AG3 than between the monocot infecting isolates AG8 and AG1-IA (Fig. 1). The two approaches combined provide strong support for the identified proteins to be distinct between isolates.

To further investigate the putative functions of the secreted proteins, PFAM domains were mapped using the PFAM batch search server^21^ and are reported in Supplementary Data S3. The proteins conserved between the two dicot infecting isolates but absent from AG1-IA had hits to 152 different Pfam domains including many CAZy families and proteins with domains previously associated with fungal pathogenesis such as metallopeptidase and thaumatins (Supplementary Data S4). Analysis of over-representation of gene ontology (GO) terms associated with the protein set revealed several DNA damage repair and carbohydrate metabolism categories to be over-represented (Supplementary Data S4). Proteins in common between AG8 and AG1-IA but absent from AG3 had hits to 22 different Pfam domains, mostly including CAZy domains, and had over-representation of several protein folding and membrane modification functions (Supplementary Data S4).

### *Rhizoctonia solani* does not employ homologs of characterised effectors nor known effector motifs

Although AG8 is notable for causing a necrotising infection of wheat, the well characterised necrotrophic pathogens of wheat mainly reside within the Dothideomycetes^22^. To understand if the ability to infect the same hosts was conferred by conserved effectors or toxins, we searched for *R. solani* homologs of 58 experimentally validated fungal effectors including Necrosis and Ethylene inducing Proteins (NEPs) from other broad host range necrotrophs^23^, however, no proteins with substantial similarity were identified in the *R. solani* secretome. Furthermore, no motifs associated with effectors from a wide range of pathogens^24–27^ were enriched in the secretome relative to the entire proteome (Supplementary Table S2). Together, these findings suggest that *R. solani* may be employing effectors of uncharacterised classes that are distinct from those of well-studied plant pathogens and horizontal gene transfer has not played a role in host acquisition.

Recently the application of machine learning algorithms trained on functionally characterised fungal effectors to predict effectors *de-novo* produced an algorithm named EffectorP^23^. EffectorP was applied to the secretomes of the individual *R. solani* isolates and in line with other pathogens, 28.7%, 24.3% and 21.7% were predicted as effectors for AG8, AG1-IA and AG3 respectively (Supplementary Data S5). Between 46% and 65% encoded unknown proteins, however, gene ontology categories related to management of reactive oxygen species, response to pathogens, transcriptional regulation and transport processes were over-represented in the EffectorP list (Supplementary Data S5). Although no homologs of known effector nor effector motifs were found in *R. solani*, the prediction of a similar proportion of the secretome as effectors by EffectorP suggests there may be conservation of underlying characteristics of effector proteins.

### *Rhizoctonia solani* has a small proportion of predicted apoplastic effectors despite lacking haustoria-like feeding structures

One set of characteristics often associated with effectors is a small size and high content of cysteine residues. This is particularly so for effectors functioning in the apoplastic space^14^. *R. solani* is known not to produce specialised intracellular structures such as haustoria^28^ suggesting that interaction with the plant may be predominantly through the apoplast. Additionally, plant cell death is known to precede the infecting *R. solani* hyphae in some cases^29^, suggesting an unknown factor moves through the plant apoplast. Small cysteine rich (SCR) proteins comprise 12.3% of the AG8 secretome, 10.9% in AG1-IA and 7.2% in AG3. This is considerably less than the 22.6% of proteins identified as SCR proteins using similar criteria in the secretomes of two other biotrophic basidiomycete pathogens of plants, *Melampsora larici-populina* and *Puccinia graminis* f. sp. *tritici* ^30^. Furthermore, the mean percentage of cysteines in the entire secretome was 1.57% for AG8, 1.635% for AG1-IA and 1.45% for AG3, which were lower than the average of 2.1% for other fungal pathogens^12^.

### Signatures of diversifying selection are identified in chitin binding proteins and proteases

Fungal effector proteins interacting with specific plant targets often exhibit higher rates of diversifying selection, evidenced by high ratios of non-synonymous to synonymous mutations (dN/dS)^31^. Few close relatives of *R. solani* have genome sequences available and comparison of proteins with highly diverse sequences can complicate the accurate calculation of dN/dS ratios. For this reason, we focused only on proteins conserved within the entire proteomes of all three *R. solani* isolates. None of the proteins in the *R. solani* combined secretome had a whole protein dN/dS greater than one. However, four proteins in AG8 and seven proteins in AG3 had an overall dN/dS above 0.75 (Supplementary Data S3, Supplementary Data S6).

Despite the low entire protein dN/dS ratios, 55 proteins from the combined secretome had significant site-specific selection^32^ (Supplementary Data S6). Three of these proteins encode putative chitin binding proteins of the same CBM14 class as the *Cladosporum fulvum* Avr4 and *Magnaporthe oryzae* Slp1 chitin-binding proteins^33, 34^. A further 6 proteins encoded putative lysine-specific metallo-endopeptidase of the M35 class that has been implicated in both virulence and avirulence in fungal pathogens of plants^35, 36^. Other protein families previously associated with fungal virulence include aspartic proteases and an inhibitor I9 domain protein similar to a cell death inducing protein from AG1-1A. The observation of site-specific diversifying selection is consistent with regions of the genes being constrained by maintaining the function of the encoded protein while other regions are undergoing diversifying selection to avoid detection by the plant immune machinery and thus are good candidates for effectors manipulating the host.

### Hierarchical clustering of *R. solani* protein tribes identifies groups of proteins with conserved effector-like characteristics

**Figure 2:**
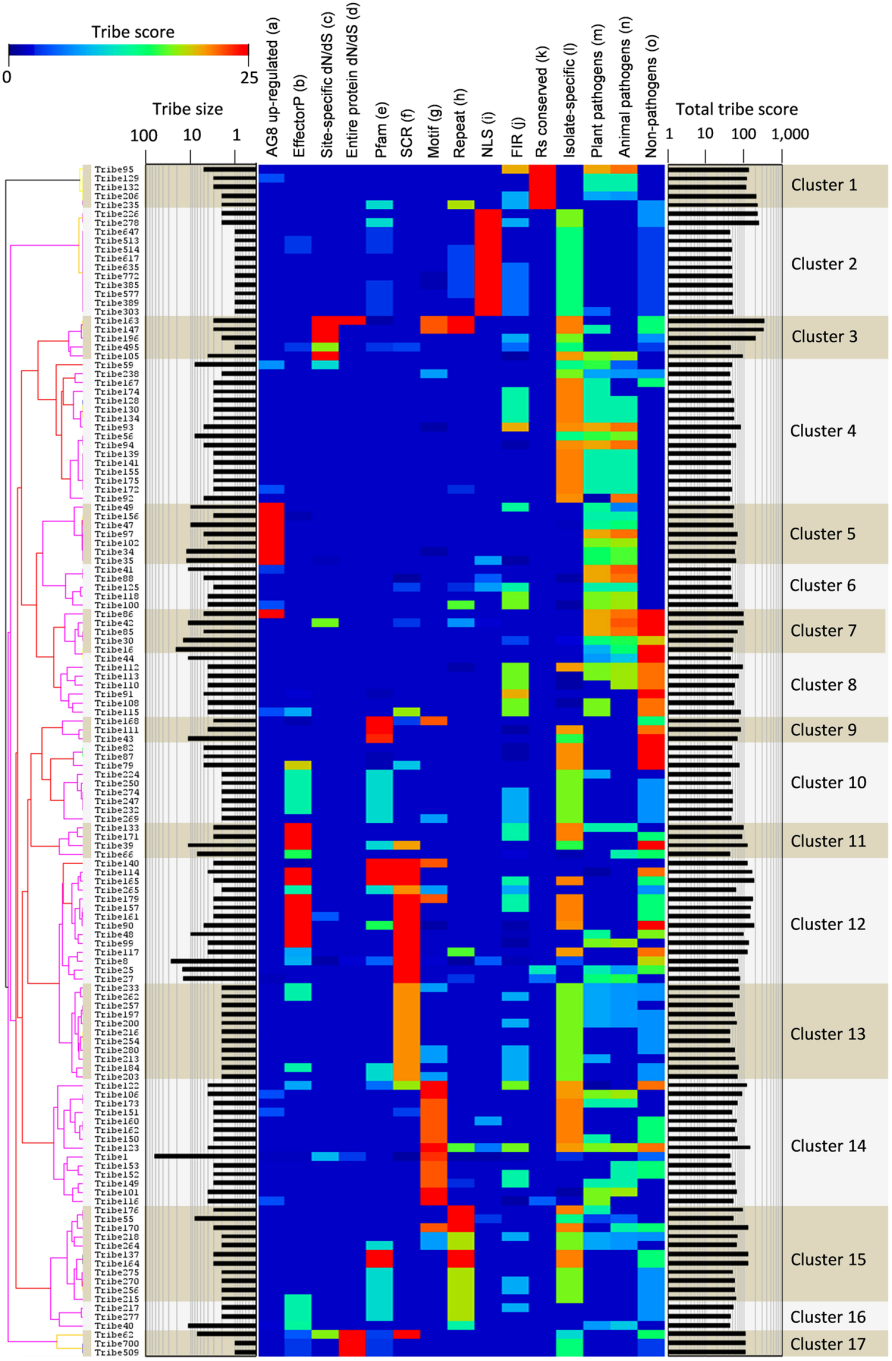
Hierarchical clustering of protein tribes reveals tribes with shared effector-like characteristics. (a–o) are heatmaps representing tribe scores for the following characteristics; (a) up-regulation of the AG8 member of the tribe during infection of wheat roots at 2 days after infection; (b) proteins predicted to be effectors by the EffectorP algorithm; (c) proteins containing site-specific diversifying selection; (d) proteins exhibiting whole protein diversifying selection; (e) annotation with a Pfam domain; (f) small cysteine rich proteins; (g) containing an effector motif-like sequence; (h) containing internal repeats; (i) containing nucleotide localisation sequences; (j) having a flanking intergenic region greater than 1 Kb; (k) conserved among AG8, AG1-IA and AG3; (l) only present in one isolate; (m) having orthologs in plant pathogenic fungi; (n) having orthologs in animal pathogenic fungi; (o) not having homologs in non-pathogenic fungi. Further details of the proteins and characteristics associated with each tribe are presented in Supplementary Data S3.

To identify tribes sharing specific characteristics and enable selection of high confidence candidate effectors from these tribes, hierarchical clustering was performed. This approach identified 17 delineated clusters and enabled selection of tribes with different sets of characteristics that may relate to different functions (Fig. 2). For example, the tribes containing a high content of SCR proteins, which together make up clusters 12 and 13, also scored highly for specificity among the *R. solani* isolates and absence from non-pathogens. Cluster 12 also had many members predicted to be effectors according to EffectorP and domains, such as CFEM, that have been previously associated with fungal pathogenesis.

The transcriptional up-regulation of pathogen genes during infection of a host plant suggests they may have a function associated with pathogenicity. Tribes with a high score for up-regulation during infection of wheat relative to vegetatively growing mycelium (Supplementary Data S3, Supplementary Table S3) typically did not possess high scores for other effector like characteristics (Cluster five). Many proteins in this cluster encode CAZys which is consistent with *in-planta* up-regulation. Moreover, the absence of other effector characteristics from CAZys is consistent with the tribe scores attributed to these proteins and supports the utility of the tribe scoring method to identify protein groups with desired characteristics.

### Inhibitor I9 domain containing proteins induce plant cell death and are more abundant in the secretomes of necrotising pathogens

**Figure 3:**
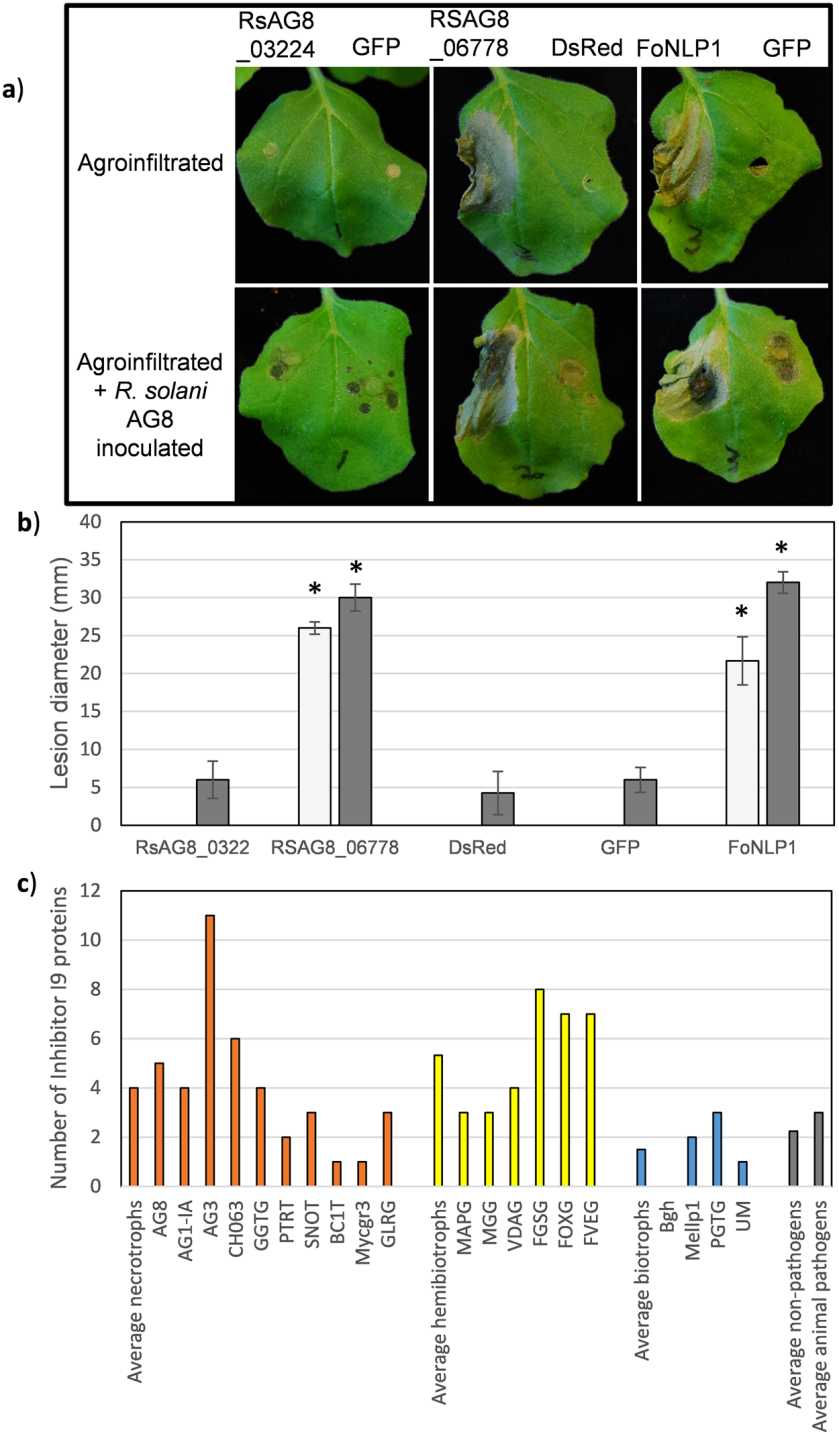
A *R. solani* AG8 Inhibitor I9 domain containing protein induces cell death when transiently expressed in *N. benthamiana* leaves. (**a**) Images acquired 5 days after agroinfiltration. (**b**) Lesion diameter on transiently transformed leaves without *R. solani* inoculation (light grey), transiently transformed leaves three days following *R. solani* AG8 inoculation (dark grey). Asterisks indicate values significantly different to the corresponding mock or *R. solani* inoculated GFP sample according to Dunnett’s test. Average values from three biological replicates is shown with standard error. The experiment was repeated three times with similar results. (**c**) Number of secreted proteins containing the Inhibitor I9 domain in fungi with different lifestyles. Abbreviations for fungal species names described in Supplementary Data S3b.

Tribe 5 (Supplementary Data S3) contains proteins with high scores for homology in plant and animal pathogens and low presence in non-pathogens. Proteins in this tribe encode protease inhibitor proteins of the I9 class including AG1IA_07795 that was previously shown to induce cell death when the protein was infiltrated into rice cultivars^17^. This tribe contains 34 members with representatives from all three isolates. To test if AG8 homologs of AG1IA_07795 possess a similar cell death inducing characteristic, the two AG8 members of tribe 5 with the highest sequence similarity to AG1IA_07795 (RsAG8_06778 and RSAG8_03224) were transiently expressed in *N. bethamiana* leaves. As a positive control for the induction of plant cell death as a result of transient expression of a fungal protein, the *Fusarium oxysporum* necrosis and ethylene inducing protein, FoNLP1, was also included our studies. Transient expression of RsAG8_06778, in *N. benthamiana* leaves caused a strong cell death response comparable to FoNLP1. The necrotic regions of leaves expressing either RsAG8_06778 or FoNPP1 supported accelerated growth of *R. solani* AG8 (Fig. 3A,B) suggesting the induced host cell death is supportive of invasion of the necrotroph rather than associated with defence factors suppressing fungal growth. The other AG8 homolog of AG1IA_07795, RSAG8_03224, was not under diversifying selection and did not induce a cell death response in *N. benthamiana*. To explore the degree of conservation of the Inhibitor I9 domain in plant pathogens, the number of secreted proteins containing the Inhibitor I9 domain (PF05922.11) was analysed for a range of fungi for which genome sequences were available and that covered a range of lifestyles. Inhibitor I9 domain containing proteins were more abundant in pathogens with a necrotising life stage (necrotrophs and hemi-biotrophs) than biotrophs, animal pathogens or non-pathogenic fungi (Fig. 3C).

### Tribe-independent selection of candidate effectors

**Figure 4:**
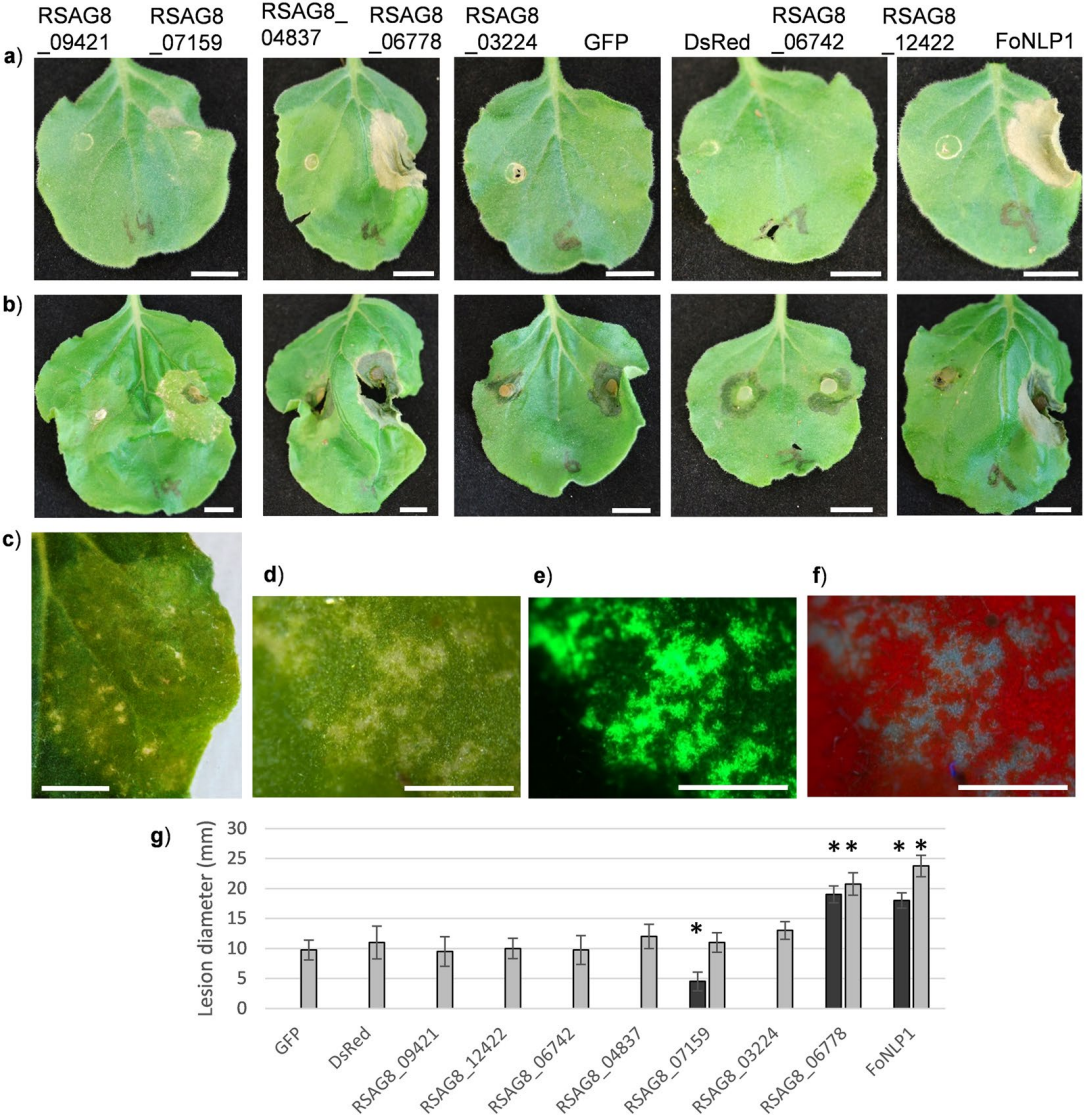
An AG8 xylanase gene induces cell death and chlorosis phenotype in *N. benthamiana*. (**a**) Images acquired 5 days after agroinfiltration. (**b**) Lesions on leaves 3 days after inoculation with *R. solani* AG8. (**c**) Closer view of a region expressing RSAG8_07159. (**d**) Light microscopy of cell death regions resulting from expression of RSAG8_07159. (**e**) Fluorescence image corresponding to (**d**) showing expression of GFP from a separate open reading frame on the same construct as RSAG8_07159. (**e**) UV light image corresponding to (**d**). Red colour indicates auto-fluorescence from chlorophyll, blue colour indicates fluorescence from necrotic cells. (**g**) Lesion diameter at 5 days after inoculation with *R. solani* AG8. Dark grey bars without *R. solani* inoculation, light grey bars with *R. solani* inoculation. The average and standard error of four replicates are presented. Asterisks indicate values significantly different to the corresponding mock or *R. solani* inoculated GFP sample according to Dunnett’s test. Scale bars 10 mm in (**a**) and (**b**) 5 mm in (**c**) and 1 mm in (**d** to **f**). The experiment was repeated three times with similar results.

Next, we investigated candidate effectors from small tribes that were not well represented in the hierarchical clustering approach above. Features used for selection of candidates focused on up-regulation during infection, diversifying selection and/or EffectorP score and selection of proteins for characterisation focused on the AG8 isolate for which we have established pathosystems. This approach identified A) 16 AG8 proteins up-regulated with a high EffectorP score B) four AG8 proteins with high EffectorP scores and undergoing diversifying selection and C) three AG8 proteins up-regulated and predicted to be undergoing diversifying selection as potential effectors. One protein from group A (high EffectorP score and up-regulated during infection) was the thaumatin protein RsAG8_08836 that our previous studies identified to be able to enhance susceptibility of *N. benthamiana* leaves to subsequent infection by *R. solani* (Anderson *et al*., 2016). Two proteins from group B were tested but did not induce plant cell death nor increased susceptibility to AG8 when expressed in *N. benthamiana* leaves (Fig. 4). The group C proteins were both up-regulated during infection and undergoing diversifying selection and among this group was the Inhibitor I9 domain containing protein (RSAG8_06778) that induced a strong cell death response when expressed in *N. benthamiana* (Fig. 3). A GH3 protein also belonging to this group, RSAG8_04837, did not induce plant cell death nor enhance susceptibility to *R. solani* AG8 when transiently expressed. The third gene, RSAG8_07159, encoding a xylanase, induced a sporadic cell death and chlorosis response when transiently expressed in leaves. However, unlike the inhibitor I9 domain containing protein (RSAG8_06778), the xylanase did not support larger lesion formation following inoculation with AG8 (Fig. 4). Unfortunately, a transformation method is not available for *R. solani* AG8 so functional analysis of RSAG8_07159 through knock-out/knock-down was not possible. Overall, the plant cell death induced from transient expression of the two proteins above suggests that combining effector prediction approaches, such as diversifying selection, EffectorP and in-planta up-regulation, are powerful complimentary methods to identify novel effectors.

## Discussion

*R. solani* is a species complex containing fungal pathogens that causes substantial losses to all of the globes major food and fibre crops (Supplementary Table S1). Despite the importance of *R. solani* to agricultural and horticultural biosecurity, little is known about the mechanisms by which the pathogen causes disease and how these may be mitigated in crop plants. Recent genomic and proteomic studies^3, 4, 6–8, 17, 37^ have provided a launching pad for investigations into conserved and unique mechanisms of pathogenesis in this pathogen. To this end, Wibberg *et al*.^8^ compared the predicted secretome of AG2-2IIIB with that of other sequenced *R. solani* isolates providing insights into CAZy family conservation, the proportion of SCR proteins and homologs of NLPs and LysM proteins. However, many other characteristics have been described for effectors from filamentous plant pathogens and in this study we present an in-depth characterisation and comparison of the secretomes of three *R. solani* isolates with distinct host ranges; a monocot specific, a dicot specific and a broad host range isolate infecting both monocots and dicots. As well as comparing CAZy families and small cysteine rich proteins, we investigate an association with the presence of characterised effector motifs, internal repeats, Pfam domains, site-specific or whole protein level diversifying selection, length of flanking intergenic region, nuclear localisation signals, *in-planta* expression profile, machine learning based prediction of effectors, conservation in plant pathogens, animal pathogens and non-pathogens and perform hierarchical clustering of protein tribes based on these characteristics to identify isolate specific and conserved effectors, or effector characteristics, that may relate to host range differences. We functionally tested several candidate effectors and identified two new proteins able to induce plant cell death.

### *R. solani* secretomes differ from that of well characterised pathogens

Fungal plant pathogens are known to secrete proteins to manipulate the host into susceptibility^38^. The size and complexity of these secretomes differ with the lifestyle and evolutionary history of the pathogen with Lin *et al*.^19^ calculating an average of 7.4% of the proteome being predicted to be secreted in 17 plant pathogenic fungi when using the same secretome prediction method as in this study. The smaller than average secretome identified in AG8 (Table 1) is apparently at odds with the broad host range of the isolate that is able to cause disease on cereals, brassicas, legumes, solanaceous species and many others^16, 39^. This suggests that the broad host range is not achieved through the acquisition of a large number of secreted proteins in an expanded genome. Similarly, other studies have suggested that broad host range necrotrophy, for example in *Sclerotinia sclerotiorum*, may be associated with the secretion of relatively few effectors that individually have activity on a broad range of hosts^40^. However, previously described broad host range effectors such as the Necrosis and ethylene-inducing Like Proteins (NLPs) are absent from *R. solani* (this study and Wibberg *et al*.^8^). An alternative route to broad host range suggested for the endomycorrhizal symbiont Rhizophagus *irregularis* is that a small secretome may minimise the potential for triggering plant immunity following detection of secreted fungal proteins^19^. However, unlike obligate endosymbionts, the secretomes of broad host range necrotrophs may be largely influenced by often extended saprophytic stages of their lifecycles requiring an array of CAZys^41^.

Taken together, the small secretome size of some *R. solani* isolates, the absence of similarity to experimentally validated effectors from other pathogens, absence of known effector motifs, a low proportion of EffectorP candidates and the absence of homology for one third of the combined secretome not possessing significant homology in other fungal plant pathogens, strongly suggest that *R. solani* employs largely novel mechanisms to induce susceptibility in host plants. Thus in addition to potentially providing insights into strategies to minimise plant susceptibility, the study of *R. solani* effectors may broaden our understanding of the biology of fungal pathogens and their hosts.

### Secretomes of *R. solani* isolates may have differentially adapted to their host range

The three *R. solani* isolates used in this study have distinct host ranges and this was reflected in the differences in their secretomes. The pattern of conservation is reminiscent of phylogenetic analysis^42^ and whole genome comparisons^6^ suggesting that reproductive isolation between anastomosis groups has driven divergence in secretome as isolates have differentially adapted to their host range. The low conservation of secreted proteins other than CAZymes between AG8 and AG1-IA suggests different mechanisms of pathogenesis on monocots may be emplyed by the isolates. Conversley, the higher conservation between non-CAZyme proteins in the secretomes of AG8 and AG3, including domains previously associated with pathogenesis (Supplementary Data S4), provide avenues to explore conserved mechansism of pathogenesis on dicot plants.

### Conserved inhibitor I9 domain proteins elicit plant cell death and are more abundant in pathogens with a necrotising lifestyle

Tribes of the combined secretome with the broadest distribution in plant pathogens but not in non-pathogens, such as tribe 5, may contain enrichment in proteins with a role in pathogenesis. Abundant within tribe 5 were peptidase S8 (Subtilase) domain proteins and the associated inhibitor I9 domain. The S8 sub-family has been associated with virulence in insect and human pathogens^43–45^ but as yet a role other than in nutrition, has not been demonstrated for plant pathogens. A member of tribe 5 from AG1-IA (AG1IA_07795) was previously shown to induce cell death when infiltrated into rice^17^ and the AG8 member undergoing diversifying selection (RsAG8_06778) was able to induce plant cell death (Fig. 3) and increase susceptibility to *R. solani* AG8 when transiently expressed in *N. benthamiana*. Given their necrotrophic lifestyle requiring death of plant cells to obtain nutrients, the presence of plant cell death inducing inhibitor I9 proteins in both AG8 and AG1-IA suggests this may be a mechanism of host manipulation conserved among *R. solani*. Furthermore, the cell death response of rice and *N. benthamiana* suggests the recognition, or the susceptibility of plants to the function of inhibitor I9 containing proteins, is broadly conserved in plants. The potential roles of this class of proteins in other plant-pathogen interactions is also supported by the expansion of the family in the secretomes of necrotrophs and hemibiotrophs (including pathogens with a significant latent period) relative to biotrophs, animal pathogens or non-pathogenic fungi (Fig. 3C). The investigation of this protein family in other fungal pathogens may shed further light on a potential broad importance of this protein family, particularly for necrotising pathogens inducing plant cell death for nutrition.

### Diversifying selection highlights proteins eliciting plant cell death

The absence of *R* genes providing resistance to *R. solani* in any host plant species suggests that PTI is likely to play a significant role in determining the outcome of plant-*R. solani* interactions. Under such circumstances, it may be anticipated that site-specific selection may be occurring on specific residues of functional proteins that are perceived by the plant and hence acting as PAMPs. In addition, negative selection pressure is likely to be occurring in functionally constrained regions of the same protein. The region-specific selection pressure caused by these opposing forces has been observed for other known PAMPs including flagellin and EF-Tu^46^. In this study, chitin binding proteins were highly represented in the list of genes undergoing site-specific diversifying selection and this is consistent with a function similar to the *Cladosporum fulvum* Avr4 CBM14 chitin binding protein, which binds chitin of the fungal cell wall to protect the fungus from plant chitinases^33^. Under these circumstances regions of the genes may be constrained by the chitin binding function while other regions are undergoing diversifying selection to avoid detection by the plant immune machinery. Another protein showing both diversifying selection and up-regulation during infection of wheat seedlings was the xylanase, RSAG8_07159 that induced plant cell death when transiently expressed in *N. benthamiana*. Plant cell death inducing xylanase proteins are involved in pathogenesis in the wheat head blight pathogen, *Fusarium graminearum* ^47^. The association of proteins undergoing site specific diversifying selection with pathogenesis and PTI suggests it may be an important feature, along with up-regulation at early stages of infection, for the identification of proteins having an effect on the host immune system. Plant germplasm screens for lines able to detect and respond to these fungal proteins or that are insensitive to the effectors cell death inducing activity may be a source of novel quantitative resistance loci for use in improving crop resistance.

## Conclusions

The in-depth characterisation and comparison of the combined secretome from three *R. solani* isolates with distinct but overlapping host-ranges provides a launch pad for investigation of pathogenicity in *R. solani*, which is of high relevance to many of the world’s largest food and fibre crops. The limited similarity between the secretomes of *R. solani* and other pathogens suggests these pathogens may employ largely novel mechanisms to induce susceptibility in plant hosts. The small secretome size of some isolates with broad host range is not associated with a large arsenal of effectors but potentially influenced by two factors, maintaining the breadth of enzymatic functions required for saprophytic life-stages and limiting opportunities for recognition of pathogen proteins as PAMPs. In this regard, hallmarks of co-evolution with a host and the expression of secretome proteins *in-planta* may be important characteristics for effector discovery in *R. solani* and other broad host range necrotrophs. Consistent with this, two novel proteins (RSAG8_06778 and RSAG8_07159) with these characteristics were found to elicit plant cell death. Furthermore, the induction of plant cell death by inhibitor I9 domain proteins may be a conserved mechanism of pathogenesis for diverse *R. solani* isolates and the potential expansion of the family in necrotrophs suggests this role may be conserved more widely.

## Methods

### Prediction and analysis of the combined AG8, AG1-IA and AG3 secretome

Detailed materials and methods for the prediction and analysis of the *Rhizoctonia solani* Kühn combined secretome are provided in the Supplementary Methods. Briefly, proteins encoded by *R. solani* AG8 WAC10335^4^ and *R. solani* AG1-IA^17^ along with predicted protein sequences from a GeneMark-ES version 2^48^ gene prediction of *R. solani* AG3 Rhs1AP^3^ were used for analysis. The SignalP 2.0 neural network algorithm was used to predict the secretome from each isolate in accordance with previous reports^30, 49^. Any protein with a predicted transmembrane domain outside of the secretion sequence according to TMHMM 2.0^50^ using default parameters or a potential mitochondrial signal according to TargetP 1.1b^51^ using default parameters were removed. Similarity between the secretomes and entire proteomes of AG8, AG1-IA and AG3 was identified using Orthofinder version 0.4^20^ and over-representation of gene ontology terms associated with proteins in isolate specific or shared orthology groups was analysed using Blast2GO^52^.

CD-HIT^53^ with the parameters –c 90 –n 5 was used to cluster highly similar proteins from all isolates with one representative selected to represent the cluster within the combined secretome. Where possible an AG8 protein was selected to represent the clustered proteins to facilitate the integration of RNAseq data. The remaining non-redundant proteins comprised the combined AG8, AG1-IA and AG3 secretome and were analysed for effector like characteristics as previously described^30^ with the addition of whole protein and site-specific dN/dS^32^, effector prediction by EffectorP v1.0^23^, fungal gene expression in infected wheat roots and the presence of homologs in fungal pathogens of plants, animal pathogens and non-pathogens as listed in Supplementary Data S3. The conservation of secreted proteins in the secretome of other fungal species was examined using BLASTp version 2.2.30. Protein hits with e-values higher than 1e-5 were discarded. Blast2GO version 6.1^52^ was used to identify gene ontology categories over-represented among groups of secretome proteins relative to the combined secretome according to Fisher’s exact test using an FDR cut-off of 0.05. Genome sequences were obtained from the BROAD Institute of Harvard and MIT (https://www.broadinstitute.org/) and the Joint Genome Institute (http://genome.jgi.doe.gov/). Secretomes were predicted according to the same method described above. The number of proteins from that species meeting the cut off was recorded for each *R. solani* secretome protein (Supplementary Data S3). Markov clustering of the combined secretome was performed using TribeMCL^54^ as previously described^55^. Although TribeMCL is good for clustering highly similar proteins into tribes, the OrthoFinder algorithm^20^ is more efficient for identifying orthologs in diverse entire genome sequences. OrthoFinder v0.4^20^ derived orthology between genes in *R. solani* isolates currently having a publicly available genome sequence is presented in Supplementary Data S7 and orthology between the AG3 protein sequences used in this study with the gene models released in Cubeta *et al*.^3^ is provided in Supplementary Data S8. Hierarchical clustering of the combined secretome tribes was performed using the following criteria. 1) up-regulation during infection, 2) EffectorP classification as candidate effectors, 3) site-specific diversifying selection 4) whole protein dN/dS, 5) having similarity to domains in the Pfam database^21^, 6) being small cysteine rich proteins with a mature length less than 150 aa and greater than 3% cysteines, 7) containing previously described effector associated motifs, 8) containing repeats, 9) containing a likely nuclear localisation signal, 10) having a flanking intergenic region greater than 1Kb, 11) being conserved in all three *R. solani* isolates, 12) being specific to a single *R. solani* isolate, 13) having homology in the predicted secretomes of other fungal pathogens of plants, 14) having homology in fungal pathogens of animals and 15) not having homology in the predicted secretomes of the non-pathogenic fungal species tested. We also searched the combined secretome for homologs of the 58 experimentally validated fungal effectors from the EffectorP training set (http://effectorp.csiro.au/data.html) using phmmer with default thresholds^56^.

### Tribe scoring

Scoring of tribes for protein characteristics was conducted as previously described^30^. Briefly, the characteristics were converted to present or absent scores for individual proteins. Tribe characteristic scores were calculated based on the proportion of proteins within that tribe that met a particular criterion and the overall tribe score calculated as a sum of all characteristic scores. Even weighting was applied to all characteristics during hierarchical clustering of tribes. The consensus tribe hierarchical tree was derived from 1000 bootstrap runs with the Pearson correlation coefficient as distance value, and average linkage between groups using MEV 4.9^57^.

### *In-planta* AG8 gene expression analysis and functional analysis of candidate effectors

Wheat seedlings were inoculated as previously described^58^ and root tissue harvested at 48 hours inoculation. Vegetative *R. solani* mycelium was grown in potato dextrose broth as previously described^37^ and was used as the reference for differential expression analysis of *R. solani* genes. RNA was extracted from three biological replicates using Trizol (Sigma) and libraries were prepared using the TruSeq Stranded Total RNA Library Kit (Illumina). The libraries sequenced on an Illumina HiSeq. 2000 (Ramaciotti Centre for Genomics, NSW) and reads were trimmed, cleaned and aligned to the AG8 genome as previously described^4^. Reads mapping to genes were counted using HTseq-count 0.6.0^59^ using intersection mode and analysis of differential expression conducted using both EdgeR 2.4.6 (prior.df = 20) and DEseq 1.6.1^60, 61^ with both methods using FDR < 0.05. Genes predicted to be significantly differentially expressed between vegetative mycelium and infected wheat roots with greater than 2 fold change in expression by both EdgeR and DEseq were considered differentially regulated for the purposes of this study. Reverse transcription and quantitative polymerase chain reaction (QPCR) were conducted on the three biological replicates and two technical repeats according Foley *et al*.^62^ using primer sequences in Supplementary Table S4.

The coding regions for candidate effector genes minus the predicted secretion sequence were fused to the *N. tabacum* PR1 secretion sequence^63^ to enable efficient secretion from *N. benthamiana* cells. The coding region was cloned under the control of the CaMV 35*S* promoter in pK7WG2D^64^ and introduced into leaves via *Agrobacterium tumefaciens* AGL1 mediated transient transformation as previously described^65^ using primers in Supplementary Table S4 or a codon optimised sequence was synthesised. Expression of GFP from an independent ORF within pK7WG2D confirmed successful transient transformation in each leaf. Each experiment was repeated a minimum of three times with similar results.

### Data Availability

All data generated or analysed during this study are included in this published article (and its Supplementary Information files) except for RNAseq data that is available under BioProject number PRJNA371695, http://www.ncbi.nlm.nih.gov/bioproject/371695.

## Electronic supplementary material

Supplementary Information.

Supplementary Data S1

Supplementary Data S2

Supplementary Data S3

Supplementary Data S4

Supplementary Data S5

Supplementary Data S6

Supplementary Data S7

Supplementary Data S8

## References

[1] Sneh, B. Identification of Rhizoctonia species. (APS Press, 1991).

[2] Naureen Z (2015). Suppression of incidence of *Rhizoctonia solani* in rice by siderophore producing rhizobacterial strains based on competition for iron. *European Scientific*. Journal.

[3] Cubeta MA (2014). Draft genome sequence of the plant-pathogenic soil fungus *Rhizoctonia solani* anastomosis group 3 strain Rhs1AP. Genome Announcements.

[4] Hane JK, Anderson JP, Williams AH, Sperschneider J, Singh KB (2014). Genome sequencing and comparative genomics of the broad host-range pathogen *Rhizoctonia solani* AG8. PLoS Genetics.

[5] Wibberg D (2013). Establishment and interpretation of the genome sequence of the phytopathogenic fungus *Rhizoctonia solani* AG1-IB isolate 7/3/14. J Biotechnol.

[6] Wibberg D (2015). Development of a *Rhizoctonia solani* AG1-IB specific gene model enables comparative genome analyses between phytopathogenic *R. solani* AG1-IA, AG1-IB, AG3 and AG8 isolates. PLoS One.

[7] Wibberg D (2015). Improved genome sequence of the phytopathogenic fungus *Rhizoctonia solani* AG1-IB 7/3/14 as established by deep mate-pair sequencing on the MiSeq (Illumina) system. J Biotechnol.

[8] Wibberg D (2016). Genome analysis of the sugar beet pathogen *Rhizoctonia solani* AG2-2IIIB revealed high numbers in secreted proteins and cell wall degrading enzymes. BMC Genomics.

[9] Zheng A (2013). The evolution and pathogenic mechanisms of the rice sheath blight pathogen. Nat Commun.

[10] Oliva R (2010). Recent developments in effector biology of filamentous plant pathogens. Cellular Microbiology.

[11] Petre B, Kamoun S (2014). How do filamentous pathogens deliver effector proteins into plant cells?. PLoS Biology.

[12] Sperschneider J (2015). Advances and challenges in computational prediction of effectors from plant pathogenic fungi. PLoS Pathog..

[13] Garnica DP, Nemri A, Upadhyaya NM, Rathjen JP, Dodds PN (2014). The ins and outs of rust haustoria. PLoS Pathog..

[14] Stergiopoulos I, de Wit PJGM (2009). Fungal effector proteins. Annu Rev Phytopathol.

[15] Sperschneider J (2014). Diversifying selection in the wheat stem rust fungus acts predominantly on pathogen-associated gene families and reveals candidate effectors. Frontiers in Plant Science.

[16] Anderson JP, Lichtenzveig J, Oliver RP, Singh KB (2013). *Medicago truncatula* as a model host for studying legume infecting *Rhizoctonia solani* and identification of a locus affecting resistance to root canker. Plant Pathol..

[17] Zheng AP (2013). The evolution and pathogenic mechanisms of the rice sheath blight pathogen. Nat. Commun..

[18] de Assis JB (2008). Divergence between sympatric rice- and soybean-infecting populations of *Rhizoctonia solani* anastomosis group-1 IA. Phytopathology.

[19] Lin K (2014). Single nucleus genome sequencing reveals high similarity among nuclei of an endomycorrhizal fungus. PLoS Genetics.

[20] Emms DM, Kelly S (2015). OrthoFinder: solving fundamental biases in whole genome comparisons dramatically improves orthogroup inference accuracy. Genome Biol..

[21] Finn RD (2014). Pfam: the protein families database. Nucleic Acids Res.

[22] Deller S, Hammond-Kosack KE, Rudd JJ (2011). The complex interactions between host immunity and non-biotrophic fungal pathogens of wheat leaves. J Plant Physiol.

[23] Sperschneider J (2016). EffectorP: predicting fungal effector proteins from secretomes using machine learning. New Phytol.

[24] Godfrey D (2010). Powdery mildew fungal effector candidates share N-terminal Y/F/WxC-motif. BMC Genomics.

[25] Kale SD, Tyler BM (2011). Entry of oomycete and fungal effectors into plant and animal host cells. Cellular Microbiology.

[26] Kale SD (2012). Oomycete and fungal effector entry, a microbial Trojan horse. New Phytol.

[27] Win J (2012). Effector biology of plant-associated organisms: concepts and perspectives. Cold Spring Harbor Symp Quant Biol.

[28] Weinhold, A. R. & Sinclair, J. B. In R*hiz*octonia *sp*ecies*: Taxonomy, molecular biology, ecology, pathology and disease control* (eds B Sneh, S Hare, S Neate, & G Dijst) Ch. III.2, 163–174 (Kluwer Academic Publishers, 1996).

[29] Keijer, J. In *Rhizoctonia species: Taxonomy, molecular biology, ecology, pathology and disease control* (eds B. Sneh, S. Hare, S. Neate, & G. Dijst) Ch. III.1, 149–162 (Kluwer Academic Publishers, 1996).

[30] Saunders DGO (2012). Using hierarchical clustering of secreted protein families to classify and rank candidate effectors of rust fungi. PLoS One.

[31] Stukenbrock EH, McDonald BA (2009). Population genetics of fungal and oomycete effectors involved in gene-for-gene interactions. Mol Plant Microbe Interact.

[32] Yang ZH, Nielsen R (2002). Codon-substitution models for detecting molecular adaptation at individual sites along specific lineages. Mol Biol Evol.

[33] van den Burg HA, Harrison SJ, Joosten M, Vervoort J, de Wit P (2006). *Cladosporium fulvum* Avr4 protects fungal cell walls against hydrolysis by plant chitinases accumulating during infection. Mol Plant Microbe Interact.

[34] Mentlak TA (2012). Effector-mediated suppression of chitin-triggered immunity by *Magnaporthe oryzae* is necessary for rice blast disease. Plant Cell.

[35] Orbach MJ, Farrall L, Sweigard JA, Chumley FG, Valent B (2000). A telomeric avirulence gene determines efficacy for the rice blast resistance gene Pi-ta. Plant Cell.

[36] Jia Y, McAdams SA, Bryan GT, Hershey HP, Valent B (2000). Direct interaction of resistance gene and avirulence gene products confers rice blast resistance. Embo J..

[37] Anderson JP (2016). Proteomic analysis of *Rhizoctonia solani* identifies infection-specific, redox associated proteins and insight into adaptation to different plant hosts. Mol Cell Proteomics.

[38] de Jonge R, Bolton MD, Thomma B (2011). How filamentous pathogens co-opt plants: the ins and outs of fungal effectors. Curr Opin Plant Biol.

[39] Anderson JP (2010). Plants versus pathogens: an evolutionary arms race. Funct Plant Biol.

[40] Guyon K, Balague C, Roby D, Raffaele S (2014). Secretome analysis reveals effector candidates associated with broad host range necrotrophy in the fungal plant pathogen *Sclerotinia sclerotiorum*. BMC Genomics.

[41] Lowe RGT, Howlett BJ (2012). Indifferent, affectionate, or deceitful: lifestyles and secretomes of fungi. PLoS Pathog..

[42] Gonzalez D (2016). Phylogenetic relationships of *Rhizoctonia* fungi within the *Cantharellales*. Fungal biology.

[43] Monod M (2002). Secreted proteases from pathogenic fungi. International Journal of Medical Microbiology.

[44] Bagga S, Hu G, Screen SE, St Leger RJ (2004). Reconstructing the diversification of subtilisins in the pathogenic fungus Metarhizium anisopliae. Gene.

[45] Jousson O (2004). Secreted subtilisin gene family in Trichophyton rubrum. Gene.

[46] McCann HC, Nahal H, Thakur S, Guttman DS (2012). Identification of innate immunity elicitors using molecular signatures of natural selection. Proc Natl Acad Sci U S A.

[47] Moscetti I (2013). Constitutive Expression of the Xylanase Inhibitor TAXI-III Delays Fusarium Head Blight Symptoms in Durum Wheat Transgenic Plants. Mol Plant Microbe Interact.

[48] Ter-Hovhannisyan V, Lomsadze A, Chernoff YO, Borodovsky M (2008). Gene prediction in novel fungal genomes using an ab initio algorithm with unsupervised training. Genome Res.

[49] Sperschneider J, Williams A, Hane J, Singh K, Taylor J (2015). Evaluation of secretion prediction highlights differing approaches needed for oomycete and fungal effectors. Frontiers in Plant Science.

[50] Krogh A, Larsson B, von Heijne G, Sonnhammer ELL (2001). Predicting transmembrane protein topology with a hidden Markov model: Application to complete genomes. J Mol Biol.

[51] Emanuelsson O, Nielsen H, Brunak S, von Heijne G (2000). Predicting subcellular localization of proteins based on their N-terminal amino acid sequence. J Mol Biol.

[52] Conesa A (2005). Blast2GO: a universal tool for annotation, visualization and analysis in functional genomics research. Bioinformatics.

[53] Fu LM, Niu BF, Zhu ZW, Wu ST, Li WZ (2012). CD-HIT: accelerated for clustering the next-generation sequencing data. Bioinformatics.

[54] Enright AJ, Van Dongen S, Ouzounis CA (2002). An efficient algorithm for large-scale detection of protein families. Nucleic Acids Res.

[55] Haas BJ (2009). Genome sequence and analysis of the Irish potato famine pathogen *Phytophthora infestans*. Nature.

[56] Finn RD, Clements J, Eddy SR (2011). HMMER web server: interactive sequence similarity searching. Nucleic Acids Res.

[57] Saeed AI (2003). TM4: A free, open-source system for microarray data management and analysis. BioTechniques.

[58] Foley RC, Kidd BN, Hane JK, Anderson JP, Singh KB (2016). Reactive oxygen species play a role in the infection of the necrotrophic fungi, *Rhizoctonia solani* in wheat. PLoS One.

[59] Anders S, Pyl PT, Huber W (2015). HTSeq-a Python framework to work with high-throughput sequencing data. Bioinformatics.

[60] Robinson MD, McCarthy DJ, Smyth GK (2010). EdgeR: a Bioconductor package for differential expression analysis of digital gene expression data. Bioinformatics.

[61] Anders S, Huber W (2010). Differential expression analysis for sequence count data. Genome Biol..

[62] Foley RC, Gleason CA, Anderson JP, Hamann T, Singh KB (2013). Genetic and genomic analysis of *Rhizoctonia solani* interactions with Arabidopsis; evidence of resistance mediated through NADPH oxidases. PLoS One.

[63] Hammond-Kosack KE, Harrison K, Jones JDG (1994). Developmentally-regulated cell-death on expression of the fungal avirulence gene *Avr9* in tomato seedlings carrying the disease-resistance gene *Cf-9*. Proc Natl Acad Sci USA.

[64] Karimi M, Inze D, Depicker A (2002). GATEWAY vectors for Agrobacterium-mediated plant transformation. Trends Plant Sci.

[65] Petrie JR (2010). Rapid expression of transgenes driven by seed-specific constructs in leaf tissue: DHA production. Plant Methods.

