# Supplementary material for: Comparative secretome analysis of Rhizoctonia solani isolates with different host ranges reveals unique secretomes and cell death inducing effectors
Source: Sci Rep. 2017 Sep 5;7:10410. doi: 10.1038/s41598-017-10405-y (PMC5585356; doi:10.1038/s41598-017-10405-y)
Supplement: Supplementary file 1 — Supplementary Information. [file 41598_2017_10405_MOESM1_ESM.pdf]

Comparative secretome analysis of *Rhizoctonia solani* isolates with different host ranges reveals unique secretomes and cell death inducing effectors

Jonathan P. Anderson, Jana Sperschneider, Joe Win, Brendan Kidd, Kentaro Yoshida, James Hane, Diane G.O. Saunders, Karam B. Singh

Supplementary Table S1. *R. solani* infection of major global food crops.
Top 15 food crops 2013
FAOSTAT (http://faostat3.fao.org/browse/rankings/commodities_by_regions/E)

Rank (global production)	Crop	Selected references for *R. solani* infection
1	Sugar cane	**Padmanabhan SY. 1946.** Rhizoctonia-leafspot, a new leaf disease of Sugarcane. *Current Science* **15**(12): 363 p.
2	Maize	**Zhou S, Ji Z, Zhao C, Liu Y, Li B. 2012.** Anastomosis grouping and genetic diversity of maize pathogen Rhizoctonia solani in Shandong. *Mycosystema* **31**(1): 31-39.
		**Singh A, Shahi JP. 2012.** Banded leaf and sheath blight: an emerging disease of maize (Zea mays L). *Maydica* **57**(1-4): 215-219.
		**McCormack AW, Woodhall JW, Back MA, Peters JC. 2013.** Rhizoctonia solani AG3-PT infecting maize stem bases and roots in the United Kingdom. *New Disease Reports* **27**: 22-22.
		**Dinesh R. 2012.** Foliar diseases of maize and their management. *International Journal of Plant Protection* **5**(2): 449-452.
		**Boine B, Renner A-C, Zellner M, Nechwatal J. 2014.** Quantitative methods for assessment of the impact of different crops on the inoculum density of Rhizoctonia solani AG2-2IIIB in soil. *European Journal of Plant Pathology* **140**(4): 745-756.
3	Rice	**Lee FN, Rush MC. 1983.** Rice sheath blight - a major rice disease. *Plant Disease* **67**(7): 829-832.
4	Wheat	**Okubara PA, Dickman MB, Blechl AE. 2014.** Molecular and genetic aspects of controlling the soilborne necrotrophic pathogens *Rhizoctonia* and *Pythium*. *Plant Science* **228**: 61-70.
		**Murray GM, Brennan JP. 2009.** *The current and potential costs from diseases of wheat in Australia*. BARTON, ACT: Grains Research and Development Corporation.
5	Potatoes	**Cubeta MA, Thomas E, Dean RA, Jabaji S, Neate SM, Tavantzis S, Toda T, Vilgalys R, Bharathan N, Fedorova-Abrams N, et al. 2014.** Draft genome sequence of the plant-pathogenic soil fungus *Rhizoctonia solani* anastomosis group 3 strain Rhs1AP. *Genome Announcements* **2**(5): e01072-01014.
6	Vegetables	**Sneh B. 1991.** *Identification of Rhizoctonia species*. St. Paul, Minnesota: APS Press.
7	Soybeans	**Nelson B, Helms T, Christianson T, Kural I. 1996.** Characterization and pathogenicity of Rhizoctonia from soybean. *Plant Disease* **80**(1): 74-80.
		**Liu Z, Sinclair JB. 1988.** Isolates of *Rhizoctonia* solani AG2-II pathogenic to soybeans. *Phytopathology* **78**(11): 1503-1503.
8	Cassava	**Lakshmanan P, Nair MC. 1985.** Comparative studies on the morphology and pathogenicity of four isolates of Rhizoctonia solani. *Madras Agricultural Journal* **72**(7): 388-393.
9	Sugar beet	**Yassin MA. 2013.** Interaction of Rhizoctonia solani Anastomosis Groups and Sugar Beet Cultivars. *Journal of Pure and Applied Microbiology* **7**(3): 1869-1876.
		**Strausbaugh CA, Eujayl IA, Panella LW. 2013.** Interaction of Sugar Beet Host Resistance and Rhizoctonia solani AG-2-2 IIIB Strains. *Plant Disease* **97**(9): 1175-1180.
10	Tomatoes	**Pourmahdi A, Taheri P. 2015.** Genetic Diversity of Thanatephorus cucumeris Infecting Tomato in Iran. *Journal of Phytopathology* **163**(1): 19-32.
11	Barley	**Murray GM, Brennan JP. 2009.** *The current and potential costs from diseases of barley in Australia*. BARTON, ACT: Grains Research and Development Corporation.
12	Watermelons	**Hall MR, Sumner DR. 1994.** Influence of cultivar and primed or germinated seed on stand establishment of watermelon in soil infested with *Pythium irregulare* or *Rhizoctonia solani* AG-4. *Crop Protection* **13**(6): 443-450.
		**Baird RE, Carling DE, Mullinix BG. 1996.** Characterization and comparison of isolates of Rhizoctonia solani AG-7 from Arkansas, Indiana, and Japan, and select AG-4 isolates. *Plant Disease* **80**(12): 1421-1424.
		**Baird RE, Carling DE. 1995.** First report of *Rhizoctonia solani* AG-7 in Indiana. *Plant Disease* **79**(3): 321-321.
		**Aiello D, Vitale A, Hyakumachi M, Polizzi G. 2012.** Molecular characterization and pathogenicity of binucleate Rhizoctonia AG-F associated to the watermelon vine decline in Italy. *European Journal of Plant Pathology* **134**(1): 161-165.
13	Bananas	**Zhang J, Wei S, Chen Y, Xie Y. 2000.** Tests on factors for the development of sheath blight in banana seedlings. *Plant Protection* **26**(2): 22-24.
		**Zhang J, Wei S, Chen Y, Xie Y. 1999.** Tests on the factors responsible for the development of banana sapling sheath blight. *Plant Protection* **25**(6): 22-24.
		**El-Deeb HM, El-Naggar MA. 2008.** Biological control of root rot disease on banana (Musa acuminate Colla) cv. Williams. *Pakistan Journal of Agriculture, Agricultural Engineering, Veterinary Sciences* **24**(2): 46-52.
		**Amani M, Avagyan G. 2014.** Isolation and identification of fungal pathogens on banana trees (Musa acuminata L.) in Iran. *International Journal of AgriScience* **4**(8): 409-413.
14	Sweet potatoes	**Ravichandran V, Sullia SB. 1983.** Pathogenic fungi from sweet potato. *Current Science* **52**(21): 1031-1031.
		**Dukes PD, Jones A. 1980.** Diseases of sweet potato seedlings. *Hortscience* **15**(3): 279-279.
15	Onions, dry	**Sharma-Poudyal D, Paulitz TC, du Toit LJ. 2015.** Evaluation of Onion Genotypes for Resistance to Stunting Caused by Rhizoctonia solani AG 8. *Hortscience* **50**(4): 551-554.
		**Sharma-Poudyal D, Paulitz TC, du Toit LJ. 2015.** Stunted Patches in Onion Bulb Crops in Oregon and Washington: Etiology and Yield Loss. *Plant Disease* **99**(5): 648-658.
		**Patzek LJ, du Toit LJ, Paulitz TC, Jones SS. 2013.** Stunting of Onion in the Columbia Basin of Oregon and Washington Caused by Rhizoctonia spp. *Plant Disease* **97**(12): 1626-1635.

Supplementary Table S2. Frequency of effector motifs in secretome and whole proteome.

	CH.C	LIAR	RXLR	YXSL[RK]	[YFW].C
RsAG8 entire proteome	0.004422	0.006419	0.03709	0.008559	0.265835
RsAG8 secretome	0.001312	0.002625	0.011811	0.003937	0.328084
RsAG1-IA entire proteome	0.006102	0.008294	0.041377	0.008294	0.335685
RsAG1-IA secretome	0.004808	0.009615	0.019231	0.004808	0.365385
RsAG3 entire proteome	0.004584	0.00748	0.032429	0.006515	0.275842
RsAG3 secretome	0.00194	0.00291	0.008729	0.00194	0.401552

Supplementary Table S3. *R. solani* AG8 genes up-regulated during infection of wheat roots.

Pfam accession	hmm name	Number of genes	Gene IDs
PF03443.9	Glyco_hydro_61	10	RsAG8_05042, RsAG8_05471, RsAG8_06607, RsAG8_07354, RsAG8_07730, RsAG8_09436, RsAG8_10222, RsAG8_10237, RsAG8_10269, RsAG8_10904
--	No match	10	RsAG8_00780, RsAG8_04037, RsAG8_04842, RsAG8_05539, RsAG8_10543, RsAG8_10837, RsAG8_11923, RsAG8_12627, RsAG8_13865, RsAG8_14006
PF04616.9	Glyco_hydro_43	4	RsAG8_04176, RsAG8_04971, RsAG8_09165, RsAG8_10884
PF00331.15	Glyco_hydro_10	3	RsAG8_01443, RsAG8_07159, RsAG8_08118
PF01083.17	Cutinase	3	RsAG8_03308, RsAG8_06851, RsAG8_06853
PF07732.10	Cu-oxidase_3	3	RsAG8_02942, RsAG8_09513, RsAG8_11633
PF00188.21	CAP	2	RsAG8_02244, RsAG8_02433
PF00314.12	Thaumatin	2	RsAG8_08836, RsAG8_12659
PF00732.14	GMC_oxred_N	2	RsAG8_10347, RsAG8_11618
PF00734.13	CBM_1	2	RsAG8_00417, RsAG8_09108
PF00840.15	Glyco_hydro_7	2	RsAG8_09191, RsAG8_09541
PF01301.14	Glyco_hydro_35	2	RsAG8_07343, RsAG8_11520
PF02225.17	PA	2	RsAG8_02688, RsAG8_08039
PF02469.17	Fasciclin	2	RsAG8_13017, RsAG8_13190
PF03372.18	Exo_endo_phos	2	RsAG8_00345, RsAG8_08644
PF04389.12	Peptidase_M28	2	RsAG8_06871, RsAG8_11772
PF05922.11	Inhibitor_I9	2	RsAG8_01708, RsAG8_06778
PF09284.5	RhgB_N	2	RsAG8_05195, RsAG8_11884
PF11327.3	DUF3129	2	RsAG8_01116, RsAG8_01117
PF11578.3	DUF3237	2	RsAG8_03004, RsAG8_05944
PF13472.1	Lipase_GDSL_2	2	RsAG8_06193, RsAG8_11614
PF14521.1	Aspzincin_M35	2	RsAG8_02429, RsAG8_03956
PB004993	Pfam-B_4993	1	RsAG8_06117
PB005381	Pfam-B_5381	1	RsAG8_01163
PB005871	Pfam-B_5871	1	RsAG8_03240
PF00080.15	Sod_Cu	1	RsAG8_07318
PF00082.17	Peptidase_S8	1	RsAG8_08847
PF00085.15	Thioredoxin	1	RsAG8_10131
PF00135.23	COesterase	1	RsAG8_02729
PF00150.13	Cellulase	1	RsAG8_01142
PF00207.17	A2M	1	RsAG8_05194
PF00264.15	Tyrosinase	1	RsAG8_11715
PF00295.12	Glyco_hydro_28	1	RsAG8_11477

Supplementary Table S3. Continued

Pfam accession	hmm name	Number of genes	Gene IDs
PF00428.14	Ribosomal_60s	1	RsAG8_01115
PF00657.17	Lipase_GDSL	1	RsAG8_05074
PF00704.23	Glyco_hydro_18	1	RsAG8_03425
PF00722.16	Glyco_hydro_16	1	RsAG8_09922
PF00723.16	Glyco_hydro_15	1	RsAG8_07826
PF00933.16	Glyco_hydro_3	1	RsAG8_04837
PF01070.13	FMN_dh	1	RsAG8_09710
PF01095.14	Pectinesterase	1	RsAG8_09107
PF01370.16	Epimerase	1	RsAG8_07875
PF01522.16	Polysacc_deac_1	1	RsAG8_12443
PF01670.11	Glyco_hydro_12	1	RsAG8_08244
PF01764.20	Lipase_3	1	RsAG8_03874
PF01975.12	SurE	1	RsAG8_02645
PF02065.13	Melibiase	1	RsAG8_08262
PF02102.10	Peptidase_M35	1	RsAG8_00244
PF02244.11	Propep_M14	1	RsAG8_11399
PF02839.9	CBM_5_12	1	RsAG8_05847
PF03067.10	Chitin_bind_3	1	RsAG8_10650
PF03572.13	Peptidase_S41	1	RsAG8_03383
PF03648.9	Glyco_hydro_67N	1	RsAG8_11029
PF03743.9	TrbI	1	RsAG8_10530
PF04027.8	DUF371	1	RsAG8_11048
PF05577.7	Peptidase_S28	1	RsAG8_02622
PF07470.8	Glyco_hydro_88	1	RsAG8_03324
PF07504.8	FTP	1	RsAG8_09266
PF07510.6	DUF1524	1	RsAG8_08277
PF07745.8	Glyco_hydro_53	1	RsAG8_13329
PF09014.5	Sushi_2	1	RsAG8_11288
PF10891.3	DUF2719	1	RsAG8_05435
PF11914.3	DUF3432	1	RsAG8_04234
PF12697.2	Abhydrolase_6	1	RsAG8_05892
PF14498.1	Glyco_hyd_65N_2	1	RsAG8_10954
PF14686.1	fn3_3	1	RsAG8_04994
PF15193.1	FAM24	1	RsAG8_01609

Supplementary Table S4. Primer sequences used in this study.

**QPCR primers**		
**Gene**	**Primer 1**	**Primer 2**
RSAG8_00084	GCTCGAGACGACTCTCAAGAA	ACATCGTTAGATTGGCACTGG
RSAG8_00780	TTTGAAGCAGCTTGGGTAGAA	AATACTCCTCGCCTTCGATTC
RSAG8_01140	GCTCGGTACATTGGTGTTGTT	CAAGTTCGAGGCGTGTGTATT
RSAG8_01256	TGTTTAGCTTGTTTGGGATGC	GATCCAACAATCCCCTTGAAT
RSAG8_02968	TGGGATCCCTACCGTTTTTAC	GTCTTCATCCGAATCGTCAGA
RSAG8_03709	ATTCATTCGCTTGTTCGCTCT	GCGATCAAAGGAGTCTTTTCC
RSAG8_09154	TACAACCCGAACGACTTTGAC	AATTGGCCTGTGCAGTAAATG
RSAG8_09201	GTAGTCGATTCCAGACGCTGA	TGGAACACATTTCCCAGTTGT
RSAG8_10837	CTTCTTCTTCTTCGCCCTCCT	CAGCAGGTCTGGTACTTCTGG
RSAG8_11513	GCACTCACCTATTCTGCGGTA	GGCAAATATTGTTGACGCACT
RSAG8_11568	TCGGTTTCTCGATTCAAGTGT	TGATCTCCTTCCCCTCGTTAT
RSAG8_11832	GTTCTTTGCTCCCTTTGCTCT	GTGTCGGTATCGTTCGTGATT
RSAG8_12186	ATGCGAACTACAGCACCACTT	CATTTCAGGAGGGCAGCTAAC
RSAG8_12616	GGGACGATGACTGTGAGAAGA	TATCCTTGCAGCACTTCCACT
RSAG8_10151	GAACATTCCTGACGCCGAGA	CCAGTACCACTTCGTAGGGC
RSAG8_03224	AATGCCAAGGCCTCCTTCTC	ACCAGCAACATAAGGGGTGG
RSAG8_06778	CACCGCCACTAACACCATCT	GACTGCTGAAGCGTGAGACT
RSAG8G_00890 (Beta-catenin control)	TGGATGAGGACGAGGAGC	GGCTTGTAGTTGCTGGCG
**Gene cloning primers**		
RsAG8_03224	AGCCATGCTCCTGCTTCCATCCCAATC	AGCCTCGAGCAATATGGCCTTGAGGTTGCTGAA
RsAG8_06778	AGCCATGCTCCGGCCAACATTCCCATC	AGCCTCGAGGCCCTGAGCCAAGAGGTTGGTAGT
RsAG8_09421	GCTGCCAGTAATGCAGCATCTGAATCTGGTCATTTAAAG	ATGAAGACATAAGCCTAGGAAAGGGCCGGTGTATTC
RSAG8_04837	GCTGCCAGTAATGCACAGTCCTTCACTCCTCGTTCAT	ATGAAGACATAAGCTTAGAAGGTTCCCTGCAAGCGA
RSAG8_06742	GCTGCCAGTAATGCAATTCCTACTAGCCTAACCAAACAAAC	ATGAAGACATAAGCCTACCGAATTGTCACTGATAAAG
RSAG8_12422	GCTGCCAGTAATGCACTTCCTTCCCACATACCAAACTCA	ATGAAGACATAAGCTTAAAAAAAAAATAGGGTTCCGTT
RSAG8_07159	GGGGACAAGTTTGTACAAAAAAGCAGGCTTCAAGCTTATGTATTTCAGCGCCACAACC	GGGGACCACTTTGTACAAGAAAGCTGGGTCGGTACCTCACGACAAGGCAGAGATGACCG
Primer to add PR1b secretion signal		ATGAAGACATAATGAGAGTATTGGTTTTACTAGCATGTCTTGCGGCTGCCAGTAATGCA

Supplementary Methods.

Prediction of the *R. solani* pan secretome

Proteins encoded by *R. solani* AG8 WAC10335[1] and *R. solani* AG1-IA[2] along with predicted protein sequences (Table S2) from a GeneMark-ES version 2[3] gene prediction of *R. solani* AG3 Rhs1AP[4] were analysed by the SignalP 2.0 neural network algorithm to predict the secretome from each isolate in accordance with previous reports[5,6]. Any protein with a predicted transmembrane domain outside of the secretion sequence according to TMHMM 2.0[7] or a potential mitochondrial signal according to TargetP 1.1b[8] were removed. Predicted secretion signal sequences were removed from the proteins, any proteins with a trimmed size less than 40 amino acid residues were removed and CD-HIT[9] used to cluster redundant proteins from all isolates with the parameters –c 90 –n 5 producing efficient clustering of orthologs but minimal clustering of homologs. In instances where proteins from multiple isolates were clustered, the AG8 protein was chosen as the cluster representative to facilitate later RNA-seq analysis. Proteins in the pan-secretome were analysed for the percent cysteine residues, the presence of known effector associated motifs, the presence in internal repeats, similarity to domains in the pfam database[10], the length of the flanking intergenic region, the presence of nuclear localisation signals, whole protein and site-specific diversifying selection, EffectorP classification as candidate effectors[11], isolate specificity or conservation, conservation in other fungal plant pathogens, animal pathogens and non-pathogenic fungi, and for AG8 proteins; their regulation during infection of wheat.

The secretome was searched for the effector motifs [L/I]xAR, [R/K]CxxCx12H, [Y/F/W]xC, KFLAK, CHXC, RQHHKRxxxxxxxxxHRRHK, HVLVxxP, [F/L]xLYLALK and G[I/F/Y][A/L/S/T]R and RxLR (between amino acids 10 to 110) and YxSL[R/K] (between amino acids 60 and 80) using Perl scripts[5,12-18]. Internal repeats within the secretome proteins were predicted using T-Reks[19] and nuclear localisation signals were predicted with PredictNLS[20]. PFAM domains were mapped on proteins using the PFAM batch search server[10]. The flanking intergenic region for the coding region relating to each secretome protein was calculated using Perl scripts. EffectorP prediction was conducted on the secretome proteins according to Sperschneider et al.[11]. Calculation of entire protein and site-specific positive selection was conducted according to Sperschneider et al.[21]. Markov clustering of the pan secretome was performed using TribeMCL[22] according to Haas et al.[23]. Orthology between the AG3 protein sequences used in this study with the gene models released in Cubeta et al.[4] is provided in Supplementary Data S8. Orthology between genes in all *R. solani* isolates currently having a publically available genome sequence according to OrthoFinder v0.4[24] is presented in Supplementary Data S7.

Conservation of proteins in *R. solani* isolates and other fungal species

To explore the potential for conservation of the proteins among the three isolates, AG8, AG1-IA and AG3, the proteins comprising the secretomes and proteomes were compared using Orthofinder version 0.4[24] to group protein into orthology groups. The presence of orthology groups between isolates was compared using VENNY[25] to display shared and isolate specific orthology groups. Blast2GO[26] was used to identify overrepresented gene ontology terms associated with proteins in shared or isolate-specific orthology groups. To take a stringent approach toward identifying proteins with potential homology in the other isolates, and to examine the potential for conservation of genes between isolates that is not reflected in the predicted secretome, 3.3e6 100 bp HiSeq reads from genomic AG8 DNA (equivalent to approximately 17x genome coverage) were mapped to the coding sequences for the pan-secretome using bowtie 2.0.5[27] using the very-sensitive-local parameters. The reads per coding region counted using bedtools 2.20.1[28]. Any coding sequence with at least one read mapping was considered potentially present in that species.

The conservation of secretome proteins in the secretome of other fungal species was examined using BLASTp version 2.2.30. Proteins hits with e-values higher than 1e-5 were disregarded. Genome sequences were obtained from data of Broad Institute of Harvard and MIT, and Joint Genome Institute. Secretomes were predicted according to the same method of the prediction of *R. solani* secretomes as described above. The number of proteins from that species meeting the cut off was recorded for each *R. solani* combined-secretome protein (Supplementary Data S3).

Gene expression analysis

Three day old seedlings of wheat genotype Chinese spring were planted into vermiculite that had been pre-inoculated with *R. solani* AG8 for 1 week at 21°C. Mock treated control seedlings were planted into vermiculite without pre-inoculation with *R. solani*. At 48 hours and 7 days after inoculation, seedlings were harvested and above ground and root tissue collected separately and frozen in liquid N_2. Additional seedlings were scored for disease development at 21 days after inoculation to confirm infection occurred successfully. RNA was extracted from three biological replicates of all treatments using Trizol (Sigma) and sequenced with 100bp paired end strand specific Illumina HiSeq reads (Ramaciotti Centre for Genomics, NSW). Sequence reads were trimmed for adapters and quality and mapped to the AG8 genome sequence (NCBI bioproject number PRJNA187548) using Tophat-2.0.8b[29] according to Hane et al.[1]. Reads mapping to genes were counted using HTseq-count 0.6.0[30] and analysis of differential expression conducted using both EdgeR 2.4.6 and DEseq 1.6.1[31,32]. Genes predicted to be significantly differentially expressed with greater than 2 fold change in expression by both EdgeR and DEseq were considered differentially regulated for the purposes of this study.

Tribe scoring

Scoring of tribes for protein characteristics was conducted according to Saunders et al.[5]. Briefly, the characteristics were converted to plus or minus scores for individual proteins as follows. The degree of conservation among the three *R. solani* isolates was assessed by identifying proteins with matches in all three isolates (*R. solani* conserved) or unique to single isolates (*R. solani* unique). Homology of secretome proteins to non-pathogens was assessed by evaluating the absence of homology in the non-pathogen secretomes tested to allow for a positive score to be incorporated. Proteins with a hit to at least one plant or animal pathogen species were considered potentially conserved. Scores for up-regulation of gene expression during infection were assigned only to tribes that contained AG8 proteins according to whether those AG8 proteins were up-regulated during infection of wheat. Tribes without AG8 proteins were given an infection-related expression score of zero (minus). Tribe characteristic scores were calculated based on the proportion of proteins within that tribe that meet a particular criteria and the overall tribe score calculated as a sum of all characteristic scores. Even weighting was applied to all characteristics since selection for tribes with particular combinations of characteristics was to be conducted only after hierarchical clustering of high scoring tribes. As tribes with low overall scores were more prone to harbouring effector properties by chance, a score threshold being the median tribe score for tribes having at least three members was calculated. Tribes having an overall score greater than the calculated median (42.03) were selected for further analyses. Hierarchical clustering of tribes was conducted with the score associated to each property for proteins in tribes considered as the 'intensity' values. The consensus tribe hierarchical tree was derived from 1000 bootstrap runs with Pearson correlation coefficient as distance value, and average linkage between groups using MEV 4.9[33]. The tribe hierarchical tree and tribe characteristics were viewed using circos-0.69-3[34].

Quantitative polymerase chain reaction and functional analysis of candidate effectors

RNA extraction, cDNA production and quantitative PCR was conducted as previously described[35] using primer sequences in Table S12. Three biological replicates and two technical repeats were analysed for each treatment.
The coding regions for candidate effector genes minus the predicted secretion sequence were cloned from cDNA using primers in Table S12 or codon optimised sequence was synthesised. The tobacco PR1 secretion sequence[36] was added to the 5' end of the open reading frame to enable efficient secretion from tobacco cells. For transient expression in *N. benthamiana*, the coding region was cloned under the control of the *35S* promoter in pK7WG2D[37] and introduced into leaves via *Agrobacterium tumefaciens* AGL1 mediated transformation as previously described[38]. Expression of GFP from pK7WG2D was observed in the infiltrated regions to confirm successful transformation and transgene expression in each leaf. Each experiment was repeated a minimum of three time with similar results

References for Supplementary Methods:

1 Hane, J. K., Anderson, J. P., Williams, A. H., Sperschneider, J. & Singh, K. B. Genome sequencing and comparative genomics of the broad host-range pathogen *Rhizoctonia solani* AG8. *PLoS Genetics* **10**, e1004281, doi:10.1371/journal.pgen.1004281 (2014).

2 Zheng, A. P. *et al.* The evolution and pathogenic mechanisms of the rice sheath blight pathogen. *Nat. Commun.* **4**, 1424, doi:10.1038/ncomms2427 (2013).

3 Ter-Hovhannisyan, V., Lomsadze, A., Chernoff, Y. O. & Borodovsky, M. Gene prediction in novel fungal genomes using an ab initio algorithm with unsupervised training. *Genome Res* **18**, 1979-1990, doi:10.1101/gr.081612.108 (2008).

4 Cubeta, M. A. *et al.* Draft genome sequence of the plant-pathogenic soil fungus *Rhizoctonia solani* anastomosis group 3 strain Rhs1AP. *Genome Announcements* **2**, e01072-01014 (2014).
5 Saunders, D. G. O. *et al.* Using hierarchical clustering of secreted protein families to classify and rank candidate effectors of rust fungi. *PLoS One* **7**, e29847, doi:10.1371/journal.pone.0029847 (2012).
6 Sperschneider, J., Williams, A., Hane, J., Singh, K. & Taylor, J. Evaluation of secretion prediction highlights differing approaches needed for oomycete and fungal effectors. *Frontiers in Plant Science* **6**, 1168, doi:10.3389/fpls.2015.01168 (2015).
7 Krogh, A., Larsson, B., von Heijne, G. & Sonnhammer, E. L. L. Predicting transmembrane protein topology with a hidden Markov model: Application to complete genomes. *J Mol Biol* **305**, 567-580, doi:10.1006/jmbi.2000.4315 (2001).
8 Emanuelsson, O., Nielsen, H., Brunak, S. & von Heijne, G. Predicting subcellular localization of proteins based on their N-terminal amino acid sequence. *J Mol Biol* **300**, 1005-1016, doi:10.1006/jmbi.2000.3903 (2000).
9 Fu, L. M., Niu, B. F., Zhu, Z. W., Wu, S. T. & Li, W. Z. CD-HIT: accelerated for clustering the next-generation sequencing data. *Bioinformatics* **28**, 3150-3152, doi:10.1093/bioinformatics/bts565 (2012).
10 Finn, R. D. *et al.* Pfam: the protein families database. *Nucleic Acids Res* **42**, D222-D230, doi:10.1093/nar/gkt1223 (2014).
11 Sperschneider, J. *et al.* EffectorP: predicting fungal effector proteins from secretomes using machine learning. *New Phytol* **210**, 743-761 (2016).
12 Yoshida, K. *et al.* Association genetics reveals three novel avirulence genes from the rice blast fungal pathogen *Magnaporthe oryzae*. *Plant Cell* **21**, 1573-1591, doi:10.1105/tpc.109.066324 (2009).
13 Li, W. *et al.* The *Magnaporthe oryzae* avirulence gene avrpiz-t encodes a predicted secreted protein that triggers the immunity in rice mediated by the blast resistance gene *Piz-t*. *Mol Plant Microbe Interact* **22**, 411-420, doi:10.1094/mpmi-22-4-0411 (2009).
14 Godfrey, D. *et al.* Powdery mildew fungal effector candidates share N-terminal Y/F/WxC-motif. *BMC Genomics* **11**, 317, doi:10.1186/1471-2164-11-317 (2010).
15 Levesque, C. A. *et al.* Genome sequence of the necrotrophic plant pathogen *Pythium ultimum* reveals original pathogenicity mechanisms and effector repertoire. *Genome Biol.* **11**, R73, doi:10.1186/gb-2010-11-7-r73 (2010).
16 Catanzariti, A. M., Dodds, P. N., Lawrence, G. J., Ayliffe, M. A. & Ellis, J. G. Haustorially expressed secreted proteins from flax rust are highly enriched for avirulence elicitors. *Plant Cell* **18**, 243-256, doi:10.1105/tpc.105.035980 (2006).
17 Whisson, S. C. *et al.* A translocation signal for delivery of oomycete effector proteins into host plant cells. *Nature* **450**, 115, doi:10.1038/nature06203 (2007).
18 Kale, S. D. Oomycete and fungal effector entry, a microbial Trojan horse. *New Phytol* **193**, 874-881, doi:10.1111/j.1469-8137.2011.03968.x (2012).
19 Jorda, J. & Kajava, A. V. T-REKS: identification of Tandem REpeats in sequences with a K-meanS based algorithm. *Bioinformatics* **25**, 2632-2638, doi:10.1093/bioinformatics/btp482 (2009).
20 Nair, R. & Rost, B. Better prediction of sub-cellular localization by combining evolutionary and structural information. *Proteins-Structure Function and Genetics* **53**, 917-930, doi:10.1002/prot.10507 (2003).
21 Sperschneider, J. *et al.* Genome-wide analysis in three Fusarium pathogens identifies rapidly evolving chromosomes and genes associated with pathogenicity. *Genome Biology and Evolution* **7**, 1613-1627, doi:10.1093/gbe/evv092 (2015).
22 Enright, A. J., Van Dongen, S. & Ouzounis, C. A. An efficient algorithm for large-scale detection of protein families. *Nucleic Acids Res* **30**, 1575-1584, doi:10.1093/nar/30.7.1575 (2002).
23 Haas, B. J. *et al.* Genome sequence and analysis of the Irish potato famine pathogen *Phytophthora infestans*. *Nature* **461**, 393-398, doi:10.1038/nature08358 (2009).
24 Emms, D. M. & Kelly, S. OrthoFinder: solving fundamental biases in whole genome comparisons dramatically improves orthogroup inference accuracy. *Genome Biol.* **16**, 157, doi:10.1186/s13059-015-0721-2 (2015).
25 Oliveros, J. C. *VENNY. An interactive tool for comparing lists with Venn Diagrams*, <http://bioinfogp.cnb.csic.es/tools/venny/index.html> (2007).
26 Conesa, A. *et al.* Blast2GO: a universal tool for annotation, visualization and analysis in functional genomics research. *Bioinformatics* **21**, 3674-3676, doi:10.1093/bioinformatics/bti610 (2005).
27 Langmead, B. & Salzberg, S. L. Fast gapped-read alignment with Bowtie 2. *Nature Methods* **9**, 357-U354, doi:10.1038/nmeth.1923 (2012).

28 Quinlan, A. R. & Hall, I. M. BEDTools: a flexible suite of utilities for comparing genomic features. *Bioinformatics* **26**, 841-842, doi:10.1093/bioinformatics/btq033 (2010).
29 Kim, D. *et al.* TopHat2: accurate alignment of transcriptomes in the presence of insertions, deletions and gene fusions. *Genome Biol.* **14**, R36, doi:10.1186/gb-2013-14-4-r36 (2013).
30 Anders, S., Pyl, P. T. & Huber, W. HTSeq-a Python framework to work with high-throughput sequencing data. *Bioinformatics* **31**, 166-169, doi:10.1093/bioinformatics/btu638 (2015).
31 Robinson, M. D., McCarthy, D. J. & Smyth, G. K. EdgeR: a Bioconductor package for differential expression analysis of digital gene expression data. *Bioinformatics* **26**, 139-140, doi:10.1093/bioinformatics/btp616 (2010).
32 Anders, S. & Huber, W. Differential expression analysis for sequence count data. *Genome Biol.* **11**, R106, doi:10.1186/gb-2010-11-10-r106 (2010).
33 Saeed, A. I. *et al.* TM4: A free, open-source system for microarray data management and analysis. *BioTechniques* **34**, 374-378 (2003).
34 Krzywinski, M. *et al.* Circos: An information aesthetic for comparative genomics. *Genome Res* **19**, 1639-1645, doi:10.1101/gr.092759.109 (2009).
35 Foley, R. C., Gleason, C. A., Anderson, J. P., Hamann, T. & Singh, K. B. Genetic and genomic analysis of *Rhizoctonia solani* interactions with Arabidopsis; evidence of resistance mediated through NADPH oxidases. *PLoS One* **8**, e56814, doi:10.1371/journal.pone.0056814 (2013).
36 Hammond-Kosack, K. E., Harrison, K. & Jones, J. D. G. Developmentally-regulated cell-death on expression of the fungal avirulence gene *Avr9* in tomato seedlings carrying the disease-resistance gene *Cf-9*. *Proc Natl Acad Sci U S A* **91**, 10445-10449, doi:10.1073/pnas.91.22.10445 (1994).
37 Karimi, M., Inze, D. & Depicker, A. GATEWAY vectors for Agrobacterium-mediated plant transformation. *Trends Plant Sci* **7**, 193-195, doi:10.1016/s1360-1385(02)02251-3 (2002).
38 Petrie, J. R. *et al.* Rapid expression of transgenes driven by seed-specific constructs in leaf tissue: DHA production. *Plant Methods* **6**, 8, doi:10.1186/1746-4811-6-8 (2010).
